# Cross-Reactive T Cell Response Exists in Chronic Lymphocytic Choriomeningitis Virus Infection upon Pichinde Virus Challenge

**DOI:** 10.3390/v14102293

**Published:** 2022-10-18

**Authors:** Jasmin Mischke, Sebastian Klein, Austin Seamann, Immo Prinz, Liisa Selin, Dario Ghersi, Markus Cornberg, Anke R.M. Kraft

**Affiliations:** 1 Department of Gastroenterology, Hepatology and Endocrinology, Hannover Medical School (MHH), 30625 Hannover, Germany; 2 German Centre for Infection Research (DZIF), 38124 Braunschweig, Germany; 3 Centre for Individualised Infection Medicine (CiiM), 30625 Hannover, Germany; 4 TWINCORE, Centre for Experimental and Clinical Infection Research, 30625 Hannover, Germany; 5 School of Interdisciplinary Informatics, College of Information Science & Technology, University of Nebraska at Omaha, Omaha, NE 68182, USA; 6 Institute of Systems Immunology, University Medical Center Hamburg-Eppendorf (UKE), 20251 Hamburg, Germany; 7 Institute of Immunology, Hannover Medical School (MHH), 30625 Hannover, Germany; 8 Department of Pathology, University of Massachusetts Medical School, Worcester, MA 01605, USA

**Keywords:** heterologous immunity, LCMV, PICV, virus-specific T cells, cross-reactive T cells, sequential infection, chronic infection

## Abstract

Immunological memory to a previously encountered pathogen can influence the outcome of a sequential infection, which is called heterologous immunity. Lymphocytic choriomeningitis virus (LCMV) immune mice develop a NP205-specific T cell response that is cross-reactive to Pichinde virus infection (PICV). So far, limited data are available if cross-reactive T cell responses appear also during chronic infections with exhausted T cell responses. Exhaustion in chronic viral infections can be treated with checkpoint inhibitors, which might affect heterologous outcomes unexpectedly. The aim of this study was to investigate the cross-reactive immune response in chronic LCMV clone 13 (LCMVcl13) infection during primary PICV infection at phenotypic, functional, and T cell receptor (TCR) level. Moreover, the influence of checkpoint inhibitor therapy with αPD-L1 was investigated. Cross-reactive NP205-specific responses were present and functional in the chronic environment. Additionally, chronically infected mice were also protected from PICV mediated weight loss compared to naive PICV mice. An altered phenotype of NP205-specific T cells was detectable, but no major differences in the clonality and diversity of their TCR repertoire were observed. Checkpoint inhibitor treatment with αPD-L1 did alter chronic LCMV infection but had no major effect on heterologous immunity to PICV. Our study demonstrated that cross-reactive CD8^+^ T cells also exist in the setting of chronic infection, indicating a clinically relevant role of cross-reactive T cells in chronic infections.

## 1. Introduction

Throughout our life, our immune system is shaped by a series of infections. Previous infection with one pathogen can alter the immune response to an unrelated pathogen, which has been defined as heterologous immunity [1,2,3,4]. Cross-reactive T cell responses have been documented in numerous studies, e.g., in humans between influenza A virus (IAV) and hepatitis C virus, between IAV and Epstein-Barr virus, and within members of the flavi-, hanta-, orthomyxo- and arenavirus family [2,5,6,7,8,9]. Cross-reactive T cells received increased attention during the current COVID-19 pandemic [10,11,12,13], because protective T cell responses from one of the four other endemic coronavirus infections could potentially minimize severe courses. Initially, the relevance of cross-reactive T cell responses was systematically investigated in mouse models, e.g., between IAV and lymphocytic choriomeningitis virus (LCMV), IAV and murine cytomegalovirus (MCMV) or between LCMV and Pichinde virus (PICV). These studies have shown that cross-reactive T cell responses can result in either enhanced or diminished protective immunity, as well as altered immunopathology [2,8,14]. LCMV and PICV both belong to the family of arenaviridae with LCMV being an old world arenavirus and PICV a new world one [9]. In this interaction, a positive influence has been shown, since exposure and T cell memory formation from acute resolving LCMV-Armstrong infection results in faster viral reduction of the sequential PICV infection (LCMV+PICV) compared with mice infected with PICV alone (naive+PICV) [9]. This protection is mediated by CD8^+^ T cell responses that are cross-reactive to the two subdominant CD8^+^ T cell epitopes of LCMV NP_205–212_ (YTVKYPNL) and of PICV NP_205–212_ (YTVKFPNM) [15]. In previously naive C57Bl/6 mice, a predicted hierarchy of CD8^+^ T cell responses (immunodominant, subdominant and cryptic) can be detected during LCMV infection which are stable during the primary response, memory homeostasis and in response to homologous LCMV infection (NP396 ≥ GP33 > GP276 > NP205) [5]. This is also seen in PICV infected mice (PICV-specific CD8^+^ T cells hierarchy: NP38 > NP16 > NP205) [5]. However, in sequential heterologous PICV infection in LCMV-immune mice, the cross-reactive, previously subdominant NP205-specific CD8^+^ T cell response dominates (10–20-fold increase), whereas the dominant primary PICV NP38-specific CD8^+^ T cell response is attenuated or even becomes subdominant [5].

Whereas the relevance of cross-reactive T cells has been studied in the setting of acute resolving infection [1,2,5,8,16], only very limited information is available in the setting of chronic infections [17,18,19] and especially in chronic infections with ongoing high viral replication such as hepatitis B and hepatitis C [20,21]. Hallmarks of chronic, but not latent, viral infections, are continuous replication of the virus and exhaustion of virus-specific T cells. T cell exhaustion is defined by the loss of cytokine production (IL2, TNFα, IFNγ) after antigen-exposure, upregulation of co-regulatory molecules (e.g., PD1, CTLA4, Tim3), reduced proliferation, as well as reduction and in some cases also deletion of virus-specific T cells [22,23,24,25]. Blocking these pathways of the co-regulatory molecules, e.g., PD1/PD-L1 pathway by monoclonal antibodies, known as checkpoint-inhibitor therapy, has been shown to reinvigorate exhausted virus-specific T cell responses resulting in viral clearance [26]. This treatment is currently used in cancer therapy [27,28] and is considered for chronic viral infections [29,30].

In order to analyze the relevance of cross-reactive T cells during chronic infection, we used the well-established model of LCMV clone 13 (LCMVcl13) infection. LCMVcl13 is nearly identical to LCMV Armstrong (LCMV) with only a three amino acid difference, but no changes in the commonly investigated LCMV-specific CD8^+^ T cell epitopes [19,31,32]. C57Bl/6 mice infected with LCMVcl13 develop a chronic infection with exhausted virus-specific T cell responses [25,26].

The aim of this study was to analyze the existence of a cross-reactive T cell response between LCMV and PICV in the setting of a chronic LCMV infection, the potential influence of checkpoint inhibitor therapy and the impact on the outcome of the PICV infection. Taken together, our study demonstrated that cross-reactive CD8^+^ T cells, as described in LCMV-immune mice, are capable of responding to sequential PICV infection also in the setting of chronic LCMV infection.

## 2. Materials and Methods

### 2.1. Ethics Statement

All experiments were approved by the Lower Saxony State animal welfare (LAVES-Niedersächsisches Landesamt für Verbraucherschutz und Lebensmittelsicherheit) under project number 33.12–42502-04-16/2127 (date of approval 02 June 2016) and were performed in accordance with ethical guidelines of Medical School Hannover (MHH), Germany; the national animal protection law, and the animal experiment regulations. The highest possible ethical standards were ensured, and all efforts were made to reduce suffering of the mice.

### 2.2. Mice, Viral Infection and Treatment

For this study, 8 to 11 week old male C57Bl/6 mice were bred and housed in the central animal facility of the Hannover Medical School under specific-pathogen-free and individually ventilated cage conditions with a 12 h light/dark cycle and received diet and water *ad libitum*.

As previously described, LCMV and PICV were propagated in baby hamster kidney cells (BHK21) [5,33]. For injection, the LCMV stocks were diluted in PBS. PICV stocks were purified through a sucrose gradient.

Mice were infected either intraperitoneally with 5 × 10^4^ plaque forming units LCMV Armstrong, which are considered immune four weeks post infection, or intravenously with high-doses (2 × 10^6^ plaque forming units) LCMVcl13 to induce a chronic infection. LCMVcl13 infected mice were then either treated with αPD-L1 (200 μg, 10F.9G2, Biolegend, San Diego, CA, USA) or control treated five times intraperitoneally every third day starting at day 23 post infection [26,34]. Mice were sequentially infected intraperitoneally with 1 × 10^7^ plaque forming units PICV at day 36 post LCMV infection. As control, age-matched naive mice were used. At day 44 post LCMV infection/day 8 post PICV infection, mice were sacrificed, immune responses and viral titer were analyzed. To address the overall suppression of chronic LCMVcl13 infection without any sequential PICV infection, mice were either infected with LCMVcl13 or LCMV Armstrong or age-matched uninfected mice were investigated (Supplementary Figure S1a).

### 2.3. Synthetic Peptides and Dextramers

The synthetic peptides for the analyzed nucleoproteins (NP) LCMV NP_396–404_ (FQPQNGQFI), LCMV NP_205–212_ (YTVKYPNL), PICV NP_38–45_ (SALDFHKV) and PICV NP_205–212_ (YTVKFPNM) were purchased from ProImmune Ltd. (Oxford, UK). Dextramers were purchased from Immudex (Copenhagen, Denmark): H-2D^b^/SALDFHKV (PICV NP38), H-2K^b^/YTVKFPNM (PICV NP205) and H-2K^b^/YTVKYPNL (LCMV NP205).

### 2.4. Cell Surface, Intracellular and Dextramer Staining for Flow Cytometry

For direct ex vivo staining of cells, one flow cytometric panel was used. Single cell suspensions from spleens were prepared and the red blood cells were lysed. Single cell suspensions of 2 × 10^6^ splenocytes each were treated with FCγR-block (αCD16/32; 2.4G2; BD Bioscience, Heidelberg, Germany) to reduce unspecific binding of antibody stainings. Further, cell suspensions were stained for virus-specific T cells using Dextramer reagents (Immudex). Briefly, cells were stained with the Dextramer (NP205 or NP38) for 20 min at 4 °C, followed by additional 15 min surface antibody staining. Fluorochrome-labeled antibodies against the cell-surface antigens CD4, CD8, CD44, CD62L, CD127, KLRG1 and PD1 were used (BioLegend or eBioscience, Hatfield, UK, Supplementary Table S1). For intracellular staining with EOMES, Ki67, Tbet (Biolegend or Invitrogen, Waltham, MA, USA, Supplementary Table S1), cells were permeabilized using the True-Nuclear transcription buffer set (Biolegend) according to the manufacturer’s protocol. Flow cytometric measurements were performed with the LSRFortessa (BD Bioscience; 3 lasers and 14 colors). The flow cytometric data were collected and analyzed with the FlowJo_V10 software (BD Bioscience). All cells were gated for lymphocyte size, single cells (doublet exclusion), live cells, CD8^+^ and CD44^+^ prior to marker gating. A detailed gating strategy is shown in Supplementary Figure S2.

### 2.5. Intracellular Cytokine Measurement

Intracellular cytokine measurements were performed with a second flow cytometric panel (Panel 2) of stimulated cells, as described previously [34]. In brief, the single cell suspensions of 2×10^6^ splenocytes were stimulated with 1 μg/mL of the peptide NP396, NP205_LCMV_ or NP38 in the presence of Brefeldin A (GolgiPlug, BD Bioscience) for 4.5 h at 37 °C and 5 % CO_2_. As controls, splenocytes were incubated with α-CD3 antibody (145-2C11; eBioscience) or with media. Cells were used for flow cytometric staining as described in 2.4. with CD8 and CD44 as surface marker stainings and intracellular cytokine staining for IFNγ and TNFα (Biolegend, Supplementary Table S1) were performed after permeabilization with the True-Nuclear transcription buffer set (Biolegend) according to the manufacturer’s protocol. Flow cytometric measurement was performed with the LSRFortessa (BD Bioscience; 3 lasers and 14 colors) and analyzed with the FlowJo_V10 software (BD Bioscience). Pre-gating was done as described in *2.4.* and a detailed gating strategy is shown in Supplementary Figure S2.

### 2.6. Viral Titer Determination

LCMV titers were determined by plaque assay as described before [34]. Briefly, homogenized kidney tissue was incubated on Vero cells with ~70% confluence in a 6-well plate, whereby log_10_ dilutions of the kidney tissue were used. Staining was performed 4 days post infection with neutral red (Sigma-Aldrich, St. Louis, MO, USA); after 2 days, plaques were counted and plaque forming units per organ were calculated. As the limit of detection, 2 counted plaques (200 plaque forming units) were set.

### 2.7. Next-Generation Sequencing

For T cell receptor (TCR) repertoire analysis, splenocytes were stained with CD4, CD8, CD44, viability stain (LD) and the respective Dextramer for fluorescence-activated cell sorting (FACS). Cells were sorted for the following populations: CD4^-^CD8^+^CD44^+^NP38^+^ and CD4^-^CD8^+^CD44^+^NP205^+^. As previously described [34], the sorted cells were prepared for next-generation sequencing. In brief, the RNA was extracted from the sorted cells using the RNeasy plus microRNA extraction kit (Qiagen, Hilden, Germany), transcribed to cDNA and amplified by PCR using the SMARTer RACE cDNA amplification kit (Takara, Kusatsu, Japan) and Advantage 2PCR kit (Takara), respectively. After determining the amplicon by size on an agarose gel (2% agarose in Tris-acetate-EDTA buffer containing Gel Red Nucleic Acid Gel Stain (10,000×, Biotium, Fremont, CA, USA)), bands were cut out of the gel and the samples were extracted using the QIAquick gel extraction kit (Qiagen). All sorted samples were performed until this step, although sorted cell numbers below 500 T cells usually did not generate a gel band and had to be excluded from further analysis. This was true for seven samples, including three naive+PICV mice. Further, the extracted samples were prepared for sequencing with an index PCR (llumina, San Diego, CA, USA, according to manufacturer’s guide) and sequenced on a MiSeq (Illumina) using V2 chemistry and 150-bp paired-end sequencing.

### 2.8. Sequencing Analysis

As described before [34], for each individual sample, quality control of forward and reverse reads was performed using fastp [35]. With the MiXCR tool [36], the assembling and alignment of reads to certain TCRbeta clonotypes were determined. In order to avoid artificial diversity increases by erroneous sequences, the number of clonotypes was trimmed down by using a 96% cutoff, as described before [37]. R, as well as the R-based program VDJtools [38] and python-based tcrdist3 [39,40], were used for graphical depiction.

### 2.9. Statistics

Statistical analysis were performed using Prism 8.4.2 (GraphPad Software, La Jolla, CA, USA). Statistics are expressed as means ± standard error of the mean (SEM). Depending on the number of groups compared and the standard deviation of the data, different statistical tests were performed. Student’s t-test was used to compare two different groups and an one-way ANOVA, followed by Tukey’s multiple comparison test, was used to compare three or more groups. Differences were considered statistically significant when *p* was <0.05 (**** *p* < 0.0001; *** *p* < 0.001; ** *p* < 0.01; * *p* < 0.05).

## 3. Results

### 3.1. Diminished T Cell Responses during Sequential PICV Infection in a Setting of Chronic LCMV Infection

Previously, it was shown that chronically LCMV infected mice have diminished immune responses compared to immune mice ([26], Table 1(I)). By measuring splenocyte numbers and IFNγ^+^ CD8^+^ T cell responses after α-CD3 stimulation, showed that this diminishment carries over into the acute PICV infection response (Table 1(II)).

Although the splenocyte numbers were significantly increased from pre- to post-PICV infection in both LCMV infected groups, lower numbers in chronic environment prior to PICV infection translated into lower numbers after PICV infection (Table 1). However, the percentage of IFNγ^+^ CD8^+^ T cells after α-CD3 stimulation was only slightly increased after PICV infection in both LCMV groups, compared to the respective group prior to PICV infection, reaching no significance (Table 1). This indicates that all mice immunologically responded to subsequent PICV infection with increasing splenocyte numbers, but showed minor increases in the overall IFNγ response, measured after α-CD3 stimulation. The diminished response in the chronic setting prior to PICV infection translated to a decreased response after sequential PICV infection (LCMVcl13+PICV) compared to LCMV+PICV mice (Table 1).

Our data showed that chronically LCMVcl13+PICV infected mice had less splenocytes and lower IFNγ^+^ CD8^+^ T cells (α-CD3) compared to LCMV+PICV mice. This led to the question whether the known cross-reactive NP205-specific T cell response is also diminished in the chronic setting.

### 3.2. Cross-Reactive Epitope-Specific T Cell Responses Existed in Chronically LCMV Infected Mice upon PICV Infection

Cross-reactive NP205 and the non-cross-reactive NP38-specific T cell responses were then investigated in more detail in sequentially LCMVcl13+PICV infected mice.

**Figure 1:**
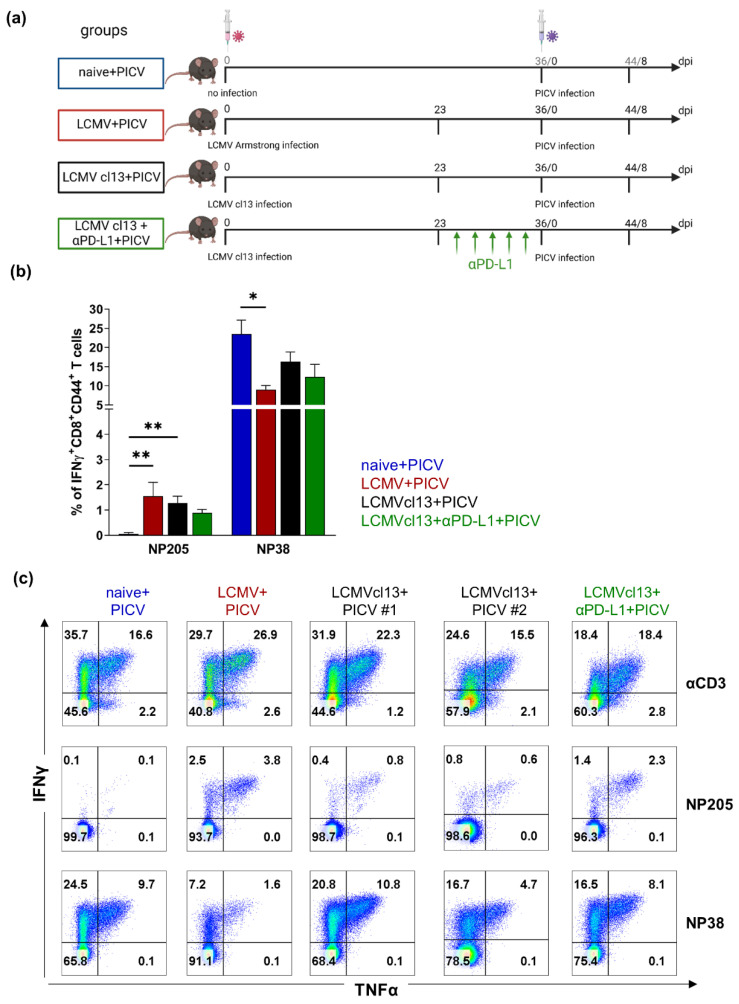
Cross-reactive NP205-specific T cells were detectable after sequential Pichinde virus (PICV) infection in chronic lymphocytic choriomeningitis virus clone 13 (LCMVcl13) mice. (**a**) Experimental setup: Age-matched C57Bl/6 mice were infected with LCMVcl13 (LCMVcl13+PICV) to induce a chronic LCMV infection, or LCMV Armstrong (LCMV+PICV), which are considered immune at day 28 post LCMV infection or kept naive (naive+PICV) prior to PICV infection. One group of LCMVcl13 mice were treated with αPD-L1 prior PICV infection (LCMVcl13+αPD-L1+PICV). (**b**) Frequency of IFNγ^+^ CD8^+^ CD44^+^ T cells after in vitro stimulation with NP205 _LCMV_ or NP38 peptides and (**c**) representative flow cytometry plots of IFNγ^+^/TNFα^+^ CD8^+^ CD44^+^ T cells after in vitro stimulation with α-CD3, NP205_LCMV_ or NP38 are shown. In (**c**), two representative mice (#1 and #2) of the LCMVcl13+PICV group are shown, accounting for inner-group diversity. Statistical comparison of all groups is depicted with asterisks. * *p* < 0.05; ** *p* < 0.01 (One-way ANOVA comparison). Results are pooled from four independent experiments with *n* = 6–14 mice/group. (**a**) was created with BioRender.com.

The cross-reactive NP205-specific T cell response was significantly increased by 27-fold in LCMV+PICV infected mice compared to mice without previous LCMV infection (naive+PICV) (Figure 1b). This is in line with published data [1,5]. The analysis of this response in chronically LCMVcl13 infected mice (LCMVcl13+PICV) also demonstrated a significantly higher frequency of the cross-reactive NP205-specific T cells (21-fold) compared to naive+PICV mice. No significant differences were observed between LCMV+PICV and LCMVcl13+PICV infected mice (Figure 1b).

In line with published data, the primary PICV, non-cross-reactive NP38-specific T cell response was significantly reduced (3-fold) in LCMV-immune compared to naive (naive+PICV) mice (Figure 1b). A reduced NP38-specific T cell response was also seen in LCMVcl13+PICV infected mice, albeit not significant.

Interestingly, in addition to the altered frequency of cross-reactive NP205-specific T cells in LCMV and LCMVcl13 infected mice, the multi-functionality of these T cells was also shifted (Figure 1c). Whereas in LCMV+PICV infected mice, all NP205 responses analyzed had higher multi-functionality (IFNγ^+^ TNFα^+^) than single IFNγ^+^ (IFNγ^+^ TNFα^-^) T cells (mean ratio: 2.3:1), this was the case in only 57 % of LCMVcl13+PICV-infected mice (representative flow cytometry plots in Figure 1c).

Our data show that cross-reactive NP205-specific T cell responses existed also in chronically LCMVcl13 infected mice upon PICV infection, but are altered and more heterogeneous.

### 3.3. Checkpoint-Inhibitor Treatment Had No Effects on the Cross-Reactive NP205-Specific T Cells in LCMVcl13+PICV Infected Mice

In the established model of chronic LCMVcl13 infection, checkpoint inhibitor therapy is effective in restoring LCMV-specific T cell responses [26,34]. Consequently, we observed enhanced IFNγ^+^ LCMV NP396-specific CD8^+^ T cells after αPD-L1 therapy compared to untreated mice, as well as increased clearance of the chronic LCMVcl13 infection, which is in line with previously published data (Supplementary Figure S1, [34]). However, αPD-L1 treatment of LCMVcl13 infected mice, prior to sequential PICV infection (LCMVcl13+αPD-L1+PICV), showed no impact on the cross-reactive NP205-specific T cell response (Figure 1).

### 3.4. Distinct Phenotype of Cross-Reactive and Non-Cross-Reactive T Cells in LCMVcl13+PICV

**Figure 2:**
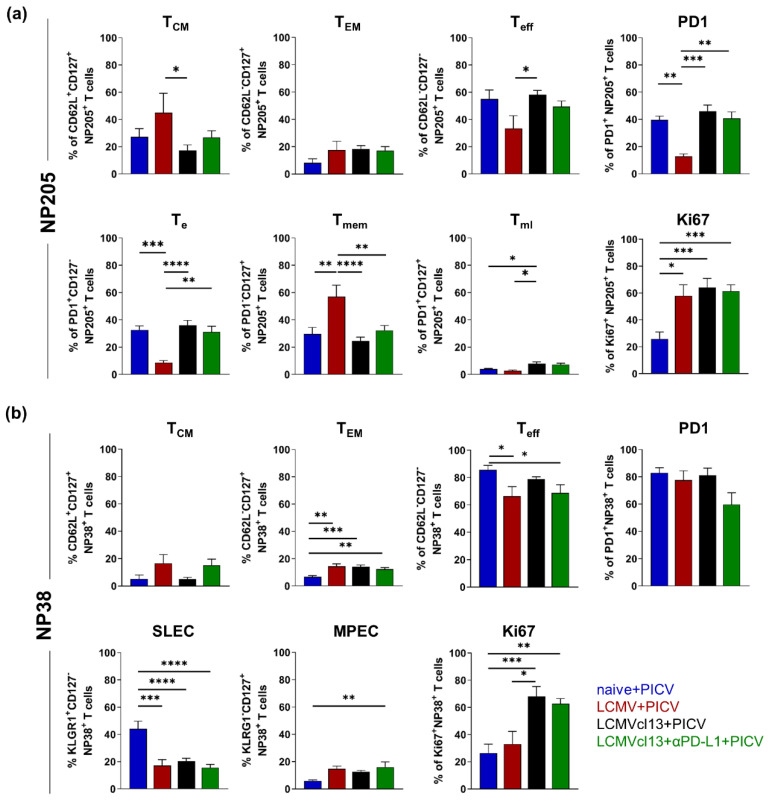
Cross-reactive NP205-specific CD8^+^ CD44^+^ T cells revealed an altered phenotype in chronic lymphocytic choriomeningitis virus clone 13 mice sequentially infected with Pichinde virus (LCMVcl13+PICV). Phenotypic characterization of (**a**) cross-reactive NP205- and (**b**) PICV NP38-Dextramer^+^ T cells were determined ex vivo. (**a**,**b**) Statistical comparison (One-way ANOVA) is depicted with asterisks. * *p* < 0.05; ** *p* < 0.01; *** *p* < 0.001; **** *p* < 0.001 Results are pooled from four independent experiments with *n* = 6–11 mice/group. T_e_: effector/terminally exhausted T cells, T_eff_: effector T cells, T_EM_: effector-memory T cells, T_ml_: memory-like T cells, T_mem_: memory T cells, T_CM_: central memory T cells, SLEC: short lived effector T cells, MPEC: memory precursor T cells.

To investigate if the cross-reactive NP205- and the non-cross-reactive NP38-specific T cell responses are phenotypically modulated in a chronic environment, we analyzed phenotypical markers on these virus-specific T cells (Figure 2a). Beside regularly used markers in a chronic setting such as PD1 and Ki67, we used different, previously published marker combinations to determine the phenotype of short-lived (CD127^−^) and long-lived (CD127^+^) T cells.

Higher frequencies of memory T cell phenotypes (T_CM_, T_EM_, T_mem_) and active cell cycle marker Ki67 were detected in the cross-reactive NP205-specific T cell response in LCMV+PICV compared to naive+PICV mice (Figure 2a). In contrast, lower frequencies of T_eff_, T_e_ and PD1^+^ T cells were measured. Higher frequencies of T_CM_ and T_mem_ memory phenotypes were also detected in LCMV+PICV compared to LCMVcl13+PICV infected mice, and in this line T_eff_, T_e_ and PD1^+^ T cells were detected in lower abundance. Albeit in very low frequencies, T_ml_ are significantly increased in LCMVcl13+PICV vs. LCMV+PICV infected mice. No differences in the frequencies of Ki67^+^ T cells were measured. Anti-PD-L1 treatment had no effect on the phenotype of the NP205-specific T cells, measured by comparing to non-treated LCMVcl13+PICV mice.

The phenotype of the primary PICV NP38-specific T cell response showed a similar alteration in all LCMV infected mice compared to the naive+PICV ones (Figure 2b). The sole exemption was the frequency of Ki67^+^ T cells, which was significantly increased in LCMVcl13+PICV compared to LCMV+PICV mice. For comparing individual groups over all phenotypical markers, radar plots are depicted in Supplementary Figure S3.

Our data suggest that phenotypical characterization in cross-reactive T cell responses in a chronic environment showed close phenotypical proximity of T cells to naive+PICV infected mice cells, while also revealing some similarities to those in LCMV+PICV infected mice. Therefore, we concluded that the chronic environment is distinctly different from both other groups, adding an additional layer of complexity to the analysis.

### 3.5. No Major Differences in the NP205-Specific TCR Repertoire between LCMV+PICV and LCMVcl13+PICV Infected Mice

**Figure 3:**
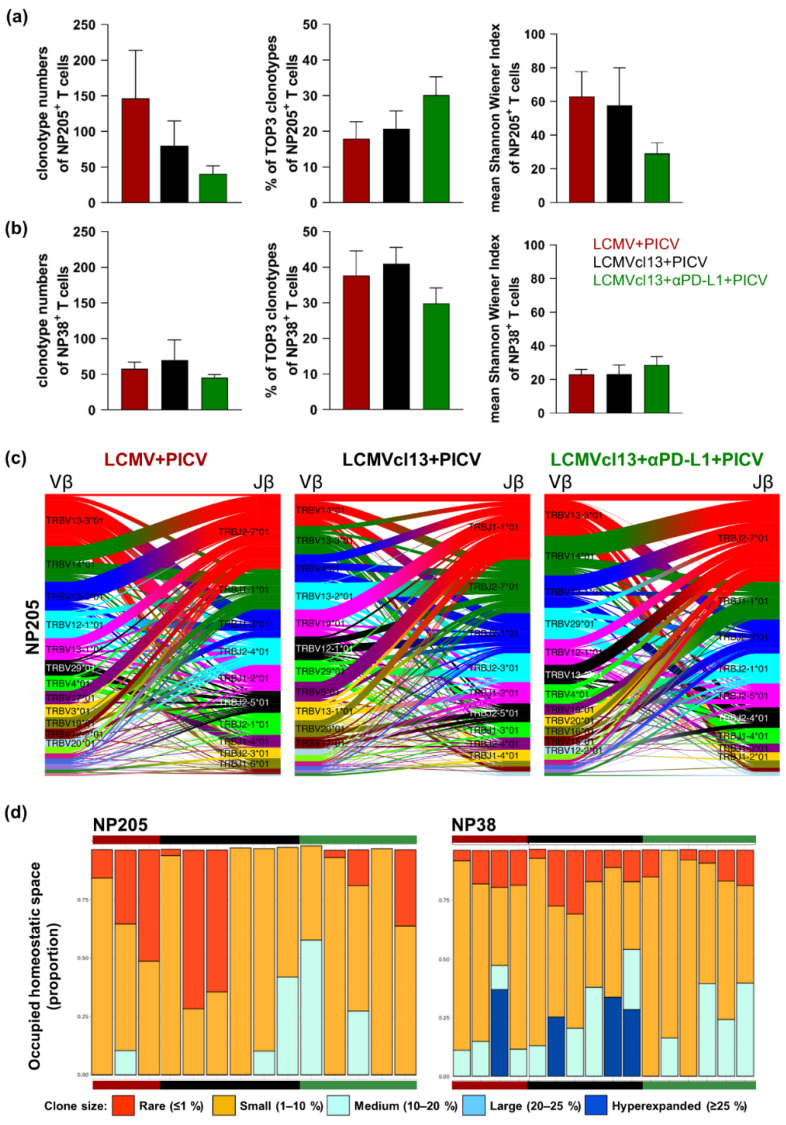
No differences were detectable in the NP205-specific T cell receptor (TCR) repertoire between chronic lymphocytic choriomeningitis virus clone 13 mice sequentially infected with Pichinde virus (LCMVcl13+PICV) and lymphocytic choriomeningitis mice sequentially infected with Pichinde virus (LCMV+PICV). Dextramer^+^ NP38- and NP205-specific T cells were sorted and TCRβ clonotypes were sequenced using the Illumina MiSeq platform. Number of (**a**) cross-reactive NP205- and (**b**) NP38-specific TCRβ clonotypes, frequency of TOP3 clonotypes and the diversity, calculated by the Shannon–Wiener index, are depicted. (**c**) Vβ and Jβ matching within the NP205-specific T cell response is depicted by lines connecting both chains, with colors from top to bottom referring to relative abundance within each individual group (ribbon plots; taking clonotype frequencies into account). (**d**) NP205- and NP38-specific TCRβ clonotypes from each individual mouse are depicted. Clonotypes were grouped in regard to their individual relative frequency: Sum of relative frequency of all hyperexpanded clonotypes (defined as ≥25% in relative frequency) are depicted in dark blue, “large” clonotypes (20 to 25%) in light blue, “medium” clonotypes (10 to 20%) in turquoise, “small” clonotypes (1 to 10%) in orange, and “rare” clonotypes (≤1%) in red. (**a**–**c**) Results are pooled from two independent experiments with *n* = 3–6 mice/group.

The TCR repertoire of LCMV NP396-specific T cells is significantly different between LCMV and LCMVcl13 mice [34]; therefore, we hypothesize that cross-reactive NP205-specific T cells might also be different prior to PICV infection and therefore translate into distinctly different TCR repertoires post PICV infection. No significant differences in the number of clonotypes, the frequency of TOP3 clonotypes, and the diversity (mean Shannon-Wiener index) were detectable for both investigated virus-specific T cell populations between the chronic setting and other groups post PICV infection (Figure 3a,b). Additionally, as control, cross-reactive NP205-specific T cells were analyzed from one naive+PICV mouse.

A narrowed TCR repertoire of NP205-specific T cells was detected in LCMV+PICV mice (Shannon–Wiener Index with mean ± SEM: 63 ± 17.8) compared to the one naive+PICV infected mouse (Shannon Wiener Index: 100.8), as also indicated by Cornberg et al. [1]. Looking at the complementary determining region 3 (CDR3) of the TCR and the Vβ, as well as Jβ usage, the NP205-specific T cells of the naive+PICV mouse has a high usage of Vβ12-1. All LCMV infected mice after PICV infection showed a similar usage of different Vβ and Jβ chains in the cross-reactive NP205-specific T cells with the usage of Vβ14 and Vβ13-3, as well as the usage of Jβ1-1 and Jβ2-7 in high frequencies (Figure 3c). In contrast, the PICV NP38-specific T cell response was altered in the different LCMV infected groups (Supplementary Figure S4). Moreover, when comparing the TCR repertoires between the NP38- and the NP205-specific T cells, significantly higher frequencies of TOP3 clonotypes of the non-cross-reactive NP38 T cells (LCMVcl13+PICV *p* = 0.01; LCMV+PICV *p* = 0.08) were in line with lower diversity ratings (LCMVcl113+PICV *p* = 0.16; LCMV+PICV *p* = 0.03) compared to the cross-reactive NP205-specific response (Figure 3). The higher diversity of the NP205-specific TCR repertoire originated from higher numbers of rare and small clonotypes compared to NP38-specific T cells, which had more high abundant (hyperexpanded) clonotypes occupying a large fraction of the repertoire (Figure 3d).

Overall, our data indicate that there are differences between the TCR repertoire of NP205- and NP38-specific responses, but no modulations occurred due to the chronic setting compared to LCMV+PICV infected mice.

### 3.6. Chronically LCMVcl13 Infected Mice Are Protected against PICV Induced Weight Loss

Additionally, to analyze the cross-reactive NP205-specific T cell response, we also investigated the disease outcome of the sequential primary PICV infection in chronically LCMVcl13 infected mice. Therefore, we closely monitored the weight of the mice after the PICV infection.

**Figure 4:**
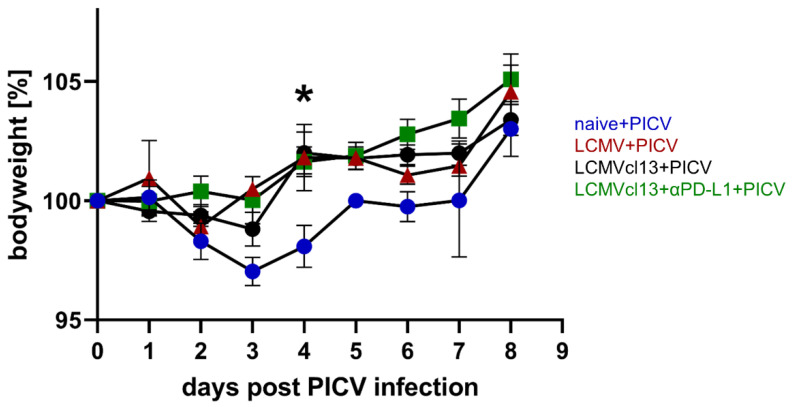
Previous lymphocytic choriomeningitis virus (LCMV) infection protected against Pichinde virus (PICV)-induced weight loss. After PICV infection, the mice were observed and weighed daily. Statistical comparisons of all mouse groups on the respective day was performed, significant difference is depicted with asterisks (* *p* < 0.05). Results are pooled from four independent experiments *n* = 6–11 mice/group.

All LCMVcl13+PICV infected mice survived without severe pathology. Interestingly, no weight loss was observed in LCMVcl13+PICV infected mice, as also seen in LCMV+PICV mice over the course of an acute PICV infection (Figure 4). A significant difference in the bodyweight was detectable at day 4 post PICV infection between naive and all previously, LCMV infected mice. In contrast, naive+PICV infected mice showed significant weight loss early after PICV infection (day 3: *p* < 0.001 and day 4: *p* = 0.0053 compared to the day of PICV infection).

Our data show that previous LCMV infection protected against PICV-induced weight loss and that cross-reactive NP205-specific T cells might be involved.

## 4. Discussion

In this study, we systematically investigated, for the first time, cross-reactive T cell responses in the setting of chronic LCMV infection in mice with and without checkpoint inhibitor therapy that were sequentially infected with PICV. Chronic LCMV infected mice were protected against PICV-induced weight loss. Additionally, these mice showed significantly more cross-reactive NP205-specific T cell responses, which are comparable to results we and others have shown in LCMV-immune mice infected with PICV [1,5,9]. Even though we found decreased numbers of splenocytes and α-CD3 induced IFNγ^+^ responses in chronically LCMV infected mice, a similar cross-reactive NP205-specific T cell response was detectable after PICV infection, compared to LCMV+PICV infected mice. Interestingly, checkpoint inhibitor therapy with αPD-L1 had no influence on the heterologous immune response.

Heterologous immunity mediated by cross-reactive T cells between unrelated viral infections has been described mainly after acute, resolving viral infections in humans and in animal models [8,16,20,41]. This has recently gained interest due to reports of cross-reactive T cell responses between endemic coronaviruses and SARS-CoV-2, which may potentially influence the course of COVID-19 [10,11]. Broadly, heterologous immunity can provide protective immunity on the one hand and trigger immunopathology on the other hand [2]. It is also known that the sequence of infections is important and can influence the existence and outcome of heterologous immunity. Whereas IAV memory response protects against vaccinia virus, it will also render hosts more susceptible to LCMV and MCMV. Interestingly, vaccinia virus immunity does not significantly affect sequential infection with LCMV or MCMV [2,42]. This phenomenon of heterologous immunity has been studied in few investigations in the context of latent viral infections, e.g., MCMV [17,18,19], but has been scarcely studied in chronic infection with ongoing high viral replication, e.g., hepatitis B, hepatitis C and LCMV [20,21,43].

Our initial hypothesis was that the immune response might be impaired in chronic LCMV infection, so that potential cross-reactive T cell responses might be absent or restricted to this extent after PICV infection, such that sequential PICV infection might have the same consequences as a PICV infection in a naive mouse. In contrast, LCMVcl13-response protected against PICV induced weight loss (Figure 4). Furthermore, we were able to detect a cross-reactive T cell response, despite LCMV-specific T cell exhaustion, in LCMVcl13+PICV infected mice (Figure 1). The data on protection are in line with data from latent MHV68 or MCMV mice sequentially infected with IAV, where enhanced survival and viral clearance has been reported [44,45]. Our data were surprising because we and others have shown strong exhaustion of virus-specific T cells in chronic viremic infections like LCMVcl13, hepatitis B virus and hepatitis C virus, which has not been reported for latent or low-level herpesvirus infections as cytomegalovirus [26,34,46,47,48,49]. However, the quality of the cross-reactive responses in the LCMVcl13 might not be similar to the LCMV-immune setting, as we could show that the CD8^+^ T cell response was generally reduced and phenotypes are different in LCMVcl13+PICV infected mice (Table 1, Figure 2).

One explanation why cross-reactive responses are present during LCMVcl13+PICV infection might be that the subdominant NP205-specific T cell responses are not as distinctly exhausted in chronic LCMVcl13, as other responses, for example the dominant NP396-specific response ([26,34], unpublished data). The first evidence that cross-reactive T cells can be detected in chronic infections was reported in patients with chronic hepatitis C virus infection, in whom cross-reactive T cells against IAV were detected [43]. However, it has not been investigated whether IAV-specific T cells have a different phenotype than in patients after resolved hepatitis C virus infection or whether these cross-reactive T cells have any clinical relevance on subsequent IAV infections. Therefore, further studies to investigate cross-reactive T cell response in chronic viremic infections in human and mouse models are needed to further understand potential beneficial effects of heterologous immunity in chronic infections.

We further investigated in this study whether checkpoint inhibitor therapy in chronically infected hosts would alter the cross-reactive T cell responses to an extent that the protective heterologous immune response would transition to immunopathology. In our LCMVcl13+PICV model we could not confirm that. An explanation for this could be that the NP205-specific T cell response is only minimally affected by checkpoint-inhibitor therapy, in contrast to the NP396-specific LCMV response [26,34]. Additional studies are needed to determine further the heterogeneity of epitope-specific T cell responses in chronic viral infections (such as NP396 vs. NP205 in chronic LCMV infection), their cross-reactive potential to an unrelated primary infection and the individual responsiveness to checkpoint inhibitor therapy.

Our study has several strengths and some limitations, as discussed below. We could establish a mouse model to investigate cross-reactive immune responses in chronic viral infections in a controlled setting, studies that are very challenging to do in humans. To our knowledge, we are the first to demonstrate cross-reactive T cell responses in chronic viral infection. Our study shows that cross-reactive T cells are still functional in a chronic environment. We hypothesize that our finding has implications also in humans, which so far remains unknown and requires further investigations. To verify this concept of cross-reactive T cells in chronically infected humans, patients chronically infected with hepatitis B virus, hepatitis C virus and human immunodeficiency viruses could be studied. Another point which needs to be considered is that cross-reactive T cells are only one part of the concept of heterologous immunity. Therefore, other mechanisms and subsets of immune cells, e.g., NK cells, B cell responses, or macrophages, as well as soluble immune factors (e.g., cytokines) that are altered in chronic infections, may also play a significant role. Viruses have been shown to be differentially susceptible to type I IFN or NK cell killing, which can be modulated by the underlying infection [2,8]. In the setting of sequential vesicular stomatitis virus (VSV) infection in chronically LCMV Docile infected mice, a prolonged type I interferon response has been shown to inhibit the enforced viral replication of VSV in specific antigen-presenting cells, such as CD169^+^ macrophages, resulting in reduced VSV titers, followed by a less strong VSV-specific immune response [18,19]. In our study, we focused on the cross-reactive CD8^+^ T cells specific for a subdominant epitope. Whether these data translate to human immune responses remains to be seen. To gain a deeper understanding of heterologous immunity in chronic viral infections, the underlying mechanisms need to be further investigated, as different chronic viral infections lead to different degrees of molecular imprinting of the immune system. Although not important in this specific model, systematic investigation of checkpoint inhibitor treatment is important in the setting because they are increasingly used to treat cancer [50].

In conclusion, we showed that a cross-reactive T cell response was detectable even in chronic viral infection and mice are protected against sequential virus-induced weight loss. Treatment with a checkpoint inhibitor had no additional effect. Our findings may provide important insight for future understanding of cross-reactive T cell responses in a chronic viral infected host and the development of, for example, virus-vector based vaccines.

## Figures and Tables

**Table 1:** The splenocyte numbers and α-CD3 response.

	Splenocyte Number			α-CD3 Response [% IFNγ^+^ CD8^+^ T Cells]		
	Naive	LCMV	LCMVcl13	Naive	LCMV	LCMVcl13
(I) no sequential infection (mean ± SEM) (*n* = 2–13)	6.5 × 10^7^ ± 1.1 × 10^7^	3.7 × 10^7^ ± 6.2 × 10^6^	2.7 × 10^7^ ± 2.7 × 10^6^	4.4 ± 0.2	47.9 ± 3.6	28.0 ± 3.5
(II) plus sequential infection (mean ± SEM) (*n* = 6–14)	8.1 × 10^7^ ± 8.3 × 10^6^	7.2 × 10^7^ ± 7.1 × 10^6^	4.3 × 10^7^ ± 6.8 × 10^6^	45.9 ± 4.2	51.3 ± 3.3	35.1 ± 2.5
*p*-value (I) vs. (II)	n.s.	0.0029	0.026	0.0019	n.s.	n.s.

## Data Availability

The data presented in this study are available on request from the corresponding author.

## Supplementary Materials

The following supporting information can be downloaded at: https://www.mdpi.com/article/10.3390/v14102293/s1, Table S1: Antibodies for flow cytometric staining; Figure S1: Checkpoint inhibitor therapy in LCMVcl13 infected mice resulted in increased LCMV NP396-specific T cell response and decreased LCMV load. Figure S2: Gating strategy. Figure S3: Phenotypical comparison of different groups from cross-reactive and non-cross-reactive T cells. Figure S4: Vβ and Jβ usage of PICV NP38-specific T cells.

## Abbreviations

IFNγ: interferon gamma, LCMV: Lymphocytic choriomeningitis virus, MPEC: memory precursor T cells (KLRG1^-^CD127^+^), NP: nucleoprotein, PICV: Pichinde virus, SEM: standard error of the mean, SLEC: short lived effector T cells (CD127^−^KLRG1^+^), TCM: central memory T cells (CD62L^+^CD127^+^), TCR: T cell receptor, Te: effector/terminally exhausted T cells (PD1^+^CD127^−^), Teff: effector T cells (CD62L^−^CD127^−^), TEM: effector-memory T cells (CD62L^−^CD127^+^), Tmem: memory T cells (PD1^-^CD127^+^), Tml: memory-like T cells (PD1^+^CD127^+^), TNFα: tumor necrosis factor alpha.

## References

[1] Cornberg M., Chen A.T., Wilkinson L.A., Brehm M.A., Kim S., Calcagno C., Ghersi D., Puzone R., Celada F., Welsh R.M. (2006). Narrowed TCR Repertoire and Viral Escape as a Consequence of Heterologous Immunity. J. Clin. Investig..

[2] Welsh R.M., Che J.W., Brehm M.A., Selin L.K. (2010). Heterologous Immunity between Viruses. Immunol. Rev..

[3] Barton E.S., White D.W., Cathelyn J.S., Brett-McClellan K.A., Engle M., Diamond M.S., Miller V.L., Virgin H.W. (2007). Herpesvirus Latency Confers Symbiotic Protection from Bacterial Infection. Nature.

[4] Sharma S., Thomas P.G. (2014). The Two Faces of Heterologous Immunity: Protection Or Immunopathology. J. Leukoc. Biol..

[5] Brehm M.A., Pinto A.K., Daniels K.A., Schneck J.P., Welsh R.M., Selin L.K. (2002). T Cell Immunodominance and Maintenance of Memory Regulated by Unexpectedly Cross-Reactive Pathogens. Nat. Immunol..

[6] Wlodarczyk M.F., Kraft A.R., Chen H.D., Kenney L.L., Selin L.K. (2013). Anti–IFN-Γ and Peptide-Tolerization Therapies Inhibit Acute Lung Injury Induced by Cross-Reactive Influenza A–Specific Memory T Cells. J. Immunol..

[7] Kraft A.R.M., Wlodarczyk M.F., Kenney L.L., Selin L.K. (2013). PC61 (Anti-CD25) Treatment Inhibits Influenza A Virus-Expanded Regulatory T Cells and Severe Lung Pathology during a Subsequent Heterologous Lymphocytic Choriomeningitis Virus Infection. J. Virol..

[8] Selin L.K., Wlodarczyk M.F., Kraft A.R., Nie S., Kenney L.L., Puzone R., Celada F. (2011). Heterologous Immunity: Immunopathology, Autoimmunity and Protection during Viral Infections. Autoimmunity.

[9] Selin L.K., Varga S.M., Wong I.C., Welsh R.M. (1998). Protective Heterologous Antiviral Immunity and Enhanced Immunopathogenesis Mediated by Memory T Cell Populations. J. Exp. Med..

[10] Lipsitch M., Grad Y.H., Sette A., Crotty S. (2020). Cross-Reactive Memory T Cells and Herd Immunity to SARS-CoV-2. Nat. Rev. Immunol..

[11] Grifoni A., Weiskopf D., Ramirez S.I., Mateus J., Dan J.M., Moderbacher C.R., Rawlings S.A., Sutherland A., Premkumar L., Jadi R.S. (2020). Targets of T Cell Responses to SARS-CoV-2 Coronavirus in Humans with COVID-19 Disease and Unexposed Individuals. Cell.

[12] Kundu R., Narean J.S., Wang L., Fenn J., Pillay T., Fernandez N.D., Conibear E., Koycheva A., Davies M., Tolosa-Wright M. (2022). Cross-Reactive Memory T Cells Associate with Protection Against SARS-CoV-2 Infection in COVID-19 Contacts. Nat. Commun..

[13] Loyal L., Braun J., Henze L., Kruse B., Dingeldey M., Reimer U., Kern F., Schwarz T., Mangold M., Unger C. (2021). Cross-Reactive CD4+ T Cells Enhance SARS-CoV-2 Immune Responses upon Infection and Vaccination. Science.

[14] Selin L.K., Brehm M.A., Naumov Y.N., Cornberg M., Kim S., Clute S.C., Welsh R.M. (2006). Memory of Mice and Men: CD8+ T-Cell Cross-Reactivity and Heterologous Immunity. Immunol. Rev..

[15] Chen A.T., Cornberg M., Gras S., Guillonneau C., Rossjohn J., Trees A., Emonet S., de la Torre J.C., Welsh R.M., Selin L.K. (2012). Loss of Anti-Viral Immunity by Infection with a Virus Encoding a Cross-Reactive Pathogenic Epitope. PLoS Pathog..

[16] Welsh R.M., Selin L.K. (2002). No One is Naive: The Significance of Heterologous T-Cell Immunity. Nat. Rev. Immunol..

[17] Che J.W., Daniels K.A., Selin L.K., Welsh R.M. (2017). Heterologous Immunity and Persistent Murine Cytomegalovirus Infection. J. Virol..

[18] Honke N., Shaabani N., Merches K., Gassa A., Kraft A., Ehrhardt K., Häussinger D., Löhning M., Dittmer U., Hengel H. (2016). Immunoactivation Induced by Chronic Viral Infection Inhibits Viral Replication and Drives Immunosuppression through Sustained IFN-I Responses. Eur. J. Immunol..

[19] Bergthaler A., Flatz L., Hegazy A.N., Johnson S., Horvath E., Löhning M., Pinschewer D.D. (2010). Viral Replicative Capacity is the Primary Determinant of Lymphocytic Choriomeningitis Virus Persistence and Immunosuppression. Proc. Natl. Acad. Sci. USA.

[20] Souquette A., Thomas P.G. (2018). Past Life and Future Effects—How Heterologous Infections Alter Immunity to Influenza Viruses. Front. Immunol..

[21] Stelekati E., Wherry E.J. (2012). Chronic Bystander Infections and Immunity to Unrelated Antigens. Cell Host Microbe.

[22] Kahan S.M., Wherry E.J., Zajac A.J. (2015). T Cell Exhaustion during Persistent Viral Infections. Virology.

[23] Wherry J.E., Blattman J.N., Murali-Krishna K., van der Most R., Ahmed R. (2003). Viral Persistence Alters CD8 T-Cell Immunodominance and Tissue Distribution and Results in Distinct Stages of Functional Impairment. J. Virol..

[24] Wherry J.E. (2011). T Cell Exhaustion. Nat. Immunol..

[25] Wherry J.E., Kurachi M. (2015). Molecular and Cellular Insights into T Cell Exhaustion. Nat. Rev. Immunol..

[26] Barber D.L., Wherry E.J., Masopust D., Zhu B., Allison J.P., Sharpe A.H., Freeman G.J., Ahmed R. (2006). Restoring Function in Exhausted CD8 T Cells during Chronic Viral Infection. Nature.

[27] Akinleye A., Rasool Z. (2019). Immune Checkpoint Inhibitors of PD-L1 as Cancer Therapeutics. J. Hematol. Oncol..

[28] Finn R.S., Qin S., Ikeda M., Galle P.R., Ducreux M., Kim T., Kudo M., Breder V., Merle P., Kaseb A.O. (2020). Atezolizumab Plus Bevacizumab in Unresectable Hepatocellular Carcinoma. N. Engl. J. Med..

[29] Gane E., Verdon D.J., Brooks A.E., Gaggar A., Nguyen A.H., Subramanian G.M., Schwabe C., Dunbar P.R. (2019). Anti-PD-1 Blockade with Nivolumab with and without Therapeutic Vaccination for Virally Suppressed Chronic Hepatitis B: A Pilot Study. J. Hepatol..

[30] A Phase 2a, Open-Label Study to Evaluate the Safety and Efficacy of Selgantolimod (SLGN)-Containing Combination Therapies for the Treatment of Chronic Hepatitis B (CHB). https://clinicaltrials.gov/ct2/show/NCT04891770.

[31] Matloubian M., Kolhekar S.R., Somasundaram T., Ahmed R. (1993). Molecular Determinants of Macrophage Tropism and Viral Persistence: Importance of Single Amino Acid Changes in the Polymerase and Glycoprotein of Lymphocytic Choriomeningitis Virus. J. Virol..

[32] Salvato M., Borrow P., Shimomaye E., Oldstone M.B. (1991). Molecular Basis of Viral Persistence: A Single Amino Acid Change in the Glycoprotein of Lymphocytic Choriomeningitis Virus is Associated with Suppression of the Antiviral Cytotoxic T-Lymphocyte Response and Establishment of Persistence. J. Virol..

[33] Welsh R.M., Seedhom M.O. (2008). Lymphocytic Choriomeningitis Virus (LCMV): Propagation, Quantitation, and Storage. Curr. Protoc. Microbiol..

[34] Klein S., Ghersi D., Manns M.P., Prinz I., Cornberg M., Kraft A.R.M. (2020). PD-L1 Checkpoint Inhibition Narrows the Antigen-Specific T Cell Receptor Repertoire in Chronic Lymphocytic Choriomeningitis Virus Infection. J. Virol..

[35] Chen S., Zhou Y., Chen Y., Gu J. (2018). Fastp: An Ultra-Fast all-in-One FASTQ Preprocessor. Bioinformatics.

[36] Bolotin D.A., Poslavsky S., Mitrophanov I., Shugay M., Mamedov I.Z., Putintseva E.V., Chudakov D.M. (2015). MiXCR: Software for Comprehensive Adaptive Immunity Profiling. Nat. Methods.

[37] Warren R.L., Freeman J.D., Zeng T., Choe G., Munro S., Moore R., Webb J.R., Holt R.A. (2011). Exhaustive T-Cell Repertoire Sequencing of Human Peripheral Blood Samples Reveals Signatures of Antigen Selection and a Directly Measured Repertoire Size of at Least 1 Million Clonotypes. Genome Res..

[38] Shugay M., Bagaev D.V., Turchaninova M.A., Bolotin D.A., Britanova O.V., Putintseva E.V., Pogorelyy M.V., Nazarov V.I., Zvyagin I.V., Kirgizova V.I. (2015). VDJtools: Unifying Post-Analysis of T Cell Receptor Repertoires. PLoS Comput. Biol..

[39] Dash P., Fiore-Gartland A.J., Hertz T., Wang G.C., Sharma S., Souquette A., Crawford J.C., Clemens E.B., Nguyen T.H.O., Kedzierska K. (2017). Quantifiable Predictive Features Define Epitope-Specific T Cell Receptor Repertoires. Nature.

[40] Gil A., Kamga L., Chirravuri-Venkata R., Aslan N., Clark F., Ghersi D., Luzuriaga K., Selin L.K. (2020). Epstein-Barr Virus Epitope-Major Histocompatibility Complex Interaction Combined with Convergent Recombination Drives Selection of Diverse T Cell Receptor A and Β Repertoires. MBio.

[41] Agrawal B. (2019). Heterologous Immunity: Role in Natural and Vaccine-Induced Resistance to Infections. Front. Immunol..

[42] Kim S., Brehm M.A., Welsh R.M., Selin L.K. (2002). Dynamics of Memory T Cell Proliferation Under Conditions of Heterologous Immunity and Bystander Stimulation. J. Immunol..

[43] Wedemeyer H., Mizukoshi E., Davis A.R., Bennink J.R., Rehermann B. (2001). Cross-Reactivity between Hepatitis C Virus and Influenza A Virus Determinant-Specific Cytotoxic T Cells. J. Virol..

[44] Saito F., Ito T., Connett J.M., Schaller M.A., Carson W.F., Hogaboam C.M., Rochford R., Kunkel S.L. (2013). MHV68 Latency Modulates the Host Immune Response to Influenza A Virus. Inflammation.

[45] Furman D., Jojic V., Sharma S., Shen-Orr S.S., Angel C.J.L., Onengut-Gumuscu S., Kidd B.A., Maecker H.T., Concannon P., Dekker C.L. (2015). Cytomegalovirus Infection Enhances the Immune Response to Influenza. Sci. Transl. Med..

[46] Aliabadi E., Urbanek-Quaing M., Maasoumy B., Bremer B., Grasshoff M., Li Y., Niehaus C.E., Wedemeyer H., Kraft A.R.M., Cornberg M. (2021). Impact of HBsAg and HBcrAg Levels on Phenotype and Function of HBV-Specific T Cells in Patients with Chronic Hepatitis B Virus Infection. Gut.

[47] Aregay A., Owusu Sekyere S., Deterding K., Port K., Dietz J., Berkowski C., Sarrazin C., Manns M.P., Cornberg M., Wedemeyer H. (2019). Elimination of Hepatitis C Virus has Limited Impact on the Functional and Mitochondrial Impairment of HCV-Specific CD8+ T Cell Responses. J. Hepatol..

[48] Owusu Sekyere S., Suneetha P.V., Hardtke S., Falk C.S., Hengst J., Manns M.P., Cornberg M., Wedemeyer H., Schlaphoff V. (2015). Type I Interferon Elevates Co-Regulatory Receptor Expression on CMV- and EBV-Specific CD8 T Cells in Chronic Hepatitis C. Front. Immunol..

[49] McLane L.M., Abdel-Hakeem M.S., Wherry E.J. (2019). CD8 T Cell Exhaustion during Chronic Viral Infection and Cancer. Annu. Rev. Immunol..

[50] Himmel M.E., Saibil S.D., Saltman A.P. (2020). Immune Checkpoint Inhibitors in Cancer Immunotherapy. CMAJ.

