# Supplementary material for: Cross-Reactive T Cell Response Exists in Chronic Lymphocytic Choriomeningitis Virus Infection upon Pichinde Virus Challenge
Source: Viruses. 2022 Oct 18;14(10):2293. doi: 10.3390/v14102293 (PMC9612015; doi:10.3390/v14102293)
Supplement: Supplementary file 1 [file viruses-14-02293-s001.zip › viruses-1927388-supplementary.pdf]

Table S1. Antibodies for flow cytometric staining.

Antibody	Company	Identifer
Rat Anti-CD4-PeCy7-conjugated (clone RM4-5)	eBioscience (Hatfield, United Kingdom)	100528
Rat Anti-CD8a-BV650-conjugated (clone 53-6.7)	Biolegend (San Diego, USA)	100741
Rat Anti-CD8a-APCFire conjugated (clone 53-6.7)	Biolegend	100766
Rat Anti-CD8a-APC eFl. 780 conjugated (clone 53-6.7)	eBioscience	47-0081-82
Rat Anti-CD44-BV510 conjugated (clone IM7)	Biolegend	103044
Rat Anti-CD62L-PerCP Cy5.5 conjugated (clone MEL-14)	Biolegend	104432
Rat Anti-CD127-BV650 conjugated (clone A7R34)	Biolegend	135043
Rat Anti-EOMES-eFluor610 conjugated (clone Dan11mag)	Invitrogen (Waltham, USA)	61-4875-82
Rat Anti-IFNγ-BV605 conjugated (clone XM61.2)	Biolegend	505840
Rat Anti-Ki67-PeCy7 conjugated (clone 16A8)	Biolegend	652425
Syrian Hamster Anti-KLRG1-BV785 conjugated (clone 2F1/KLRG1)	Biolegend	138429
Rat Anti-PD1-BV421 conjugated (clone 29F.1A12)	Biolegend	135221
Mouse Anti-Tbet-BV711 conjugated (clone 4B10)	Biolegend	644820
Rat Anti-TNFα-PeCy5 conjugated (clone MP6-XT22)	Biolegend	506322

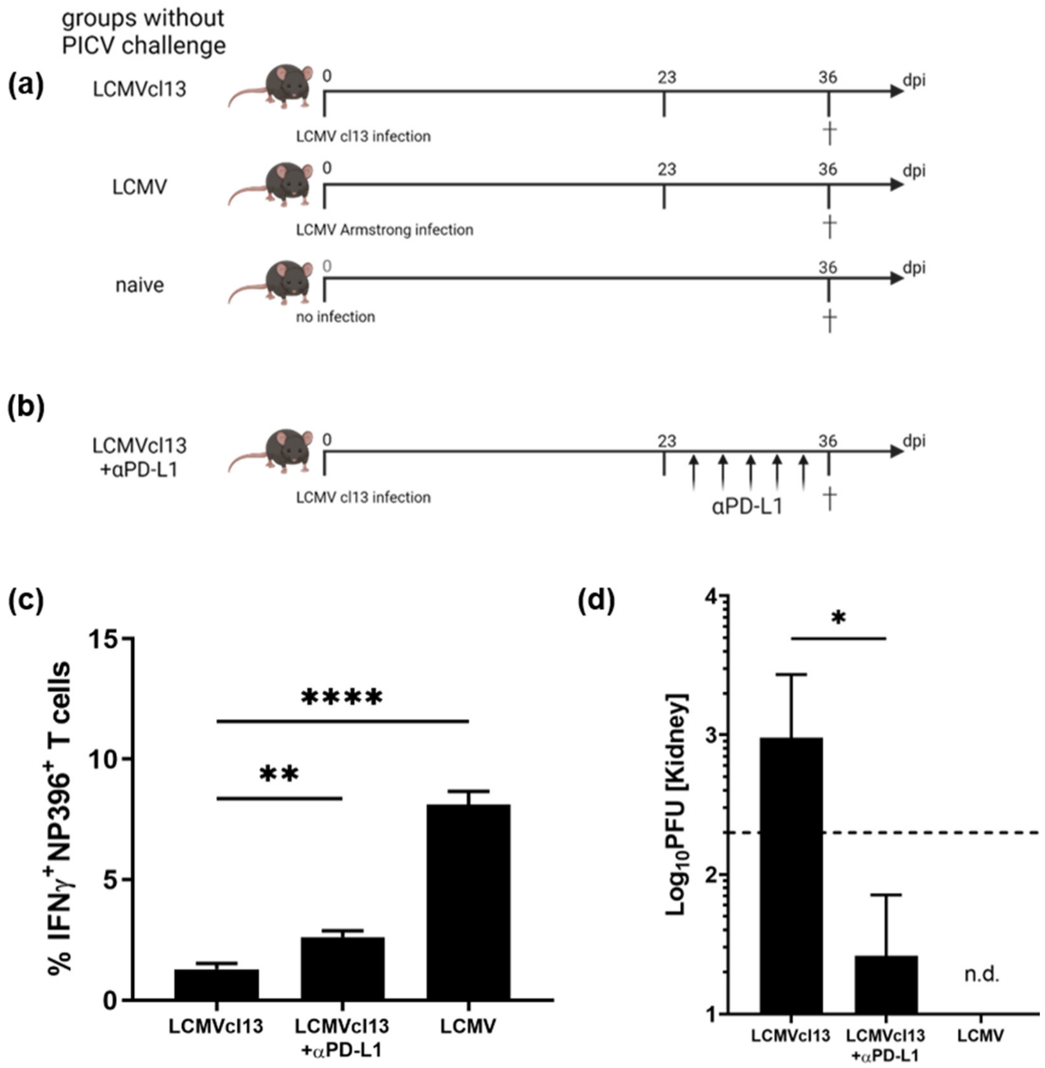

Figure S1. Checkpoint inhibitor therapy in LCMVcl13 infected mice resulted in increased LCMV NP396-specific T cell response and decreased LCMV load. (a) Experimental setup: Age-matched C57Bl/6 mice were infected with LCMV clone 13 (LCMVcl13), with LCMV Armstrong (LCMV) or kept naive (naive) without sequential PICV infection. (b) One group of LCMVcl13 mice was treated with *α*PD-L1 antibody (LCMVcl13+*α*PD-L1). (c) Frequency of IFNγ^+ CD8$^+$ T cells after stimulation with LCMV NP396 peptide. (d) LCMV load determined by plaque assay at day 36 post LCMV infection. The limit of detection is indicated by the line (n.d.: not detectable). Results are pooled from four independent experiments with n=9-16 mice/group. Statistical comparisons are depicted with asterisks. **p*=0.05; ***p*<0.01; *****p*<0.001 (One-way ANOVA). (a) was created with BioRender.com.

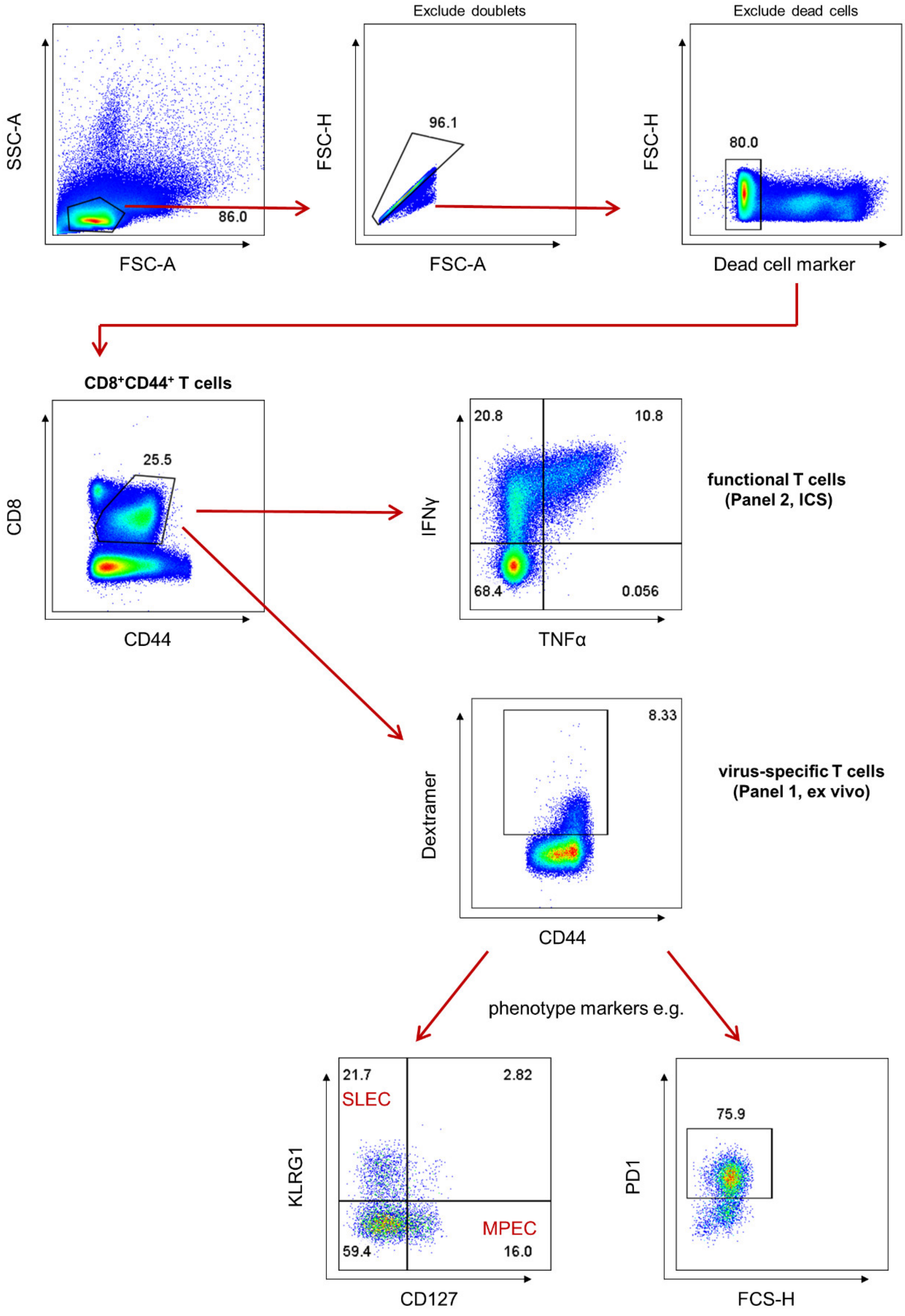

Figure S2. Gating strategy. This strategy was used to identify functional CD8+ CD44+ T cells, identified by IFNγ and TNFα, after in vitro restimulation. Virus-specific CD8+ CD44+ T cells were further phenotypically characterized ex vivo, e.g. SLEC: short lived effector T cells (CD127- KLRG1+), MPEC: memory precursor T cells (KLRG1- CD127+) and PD1 staining.

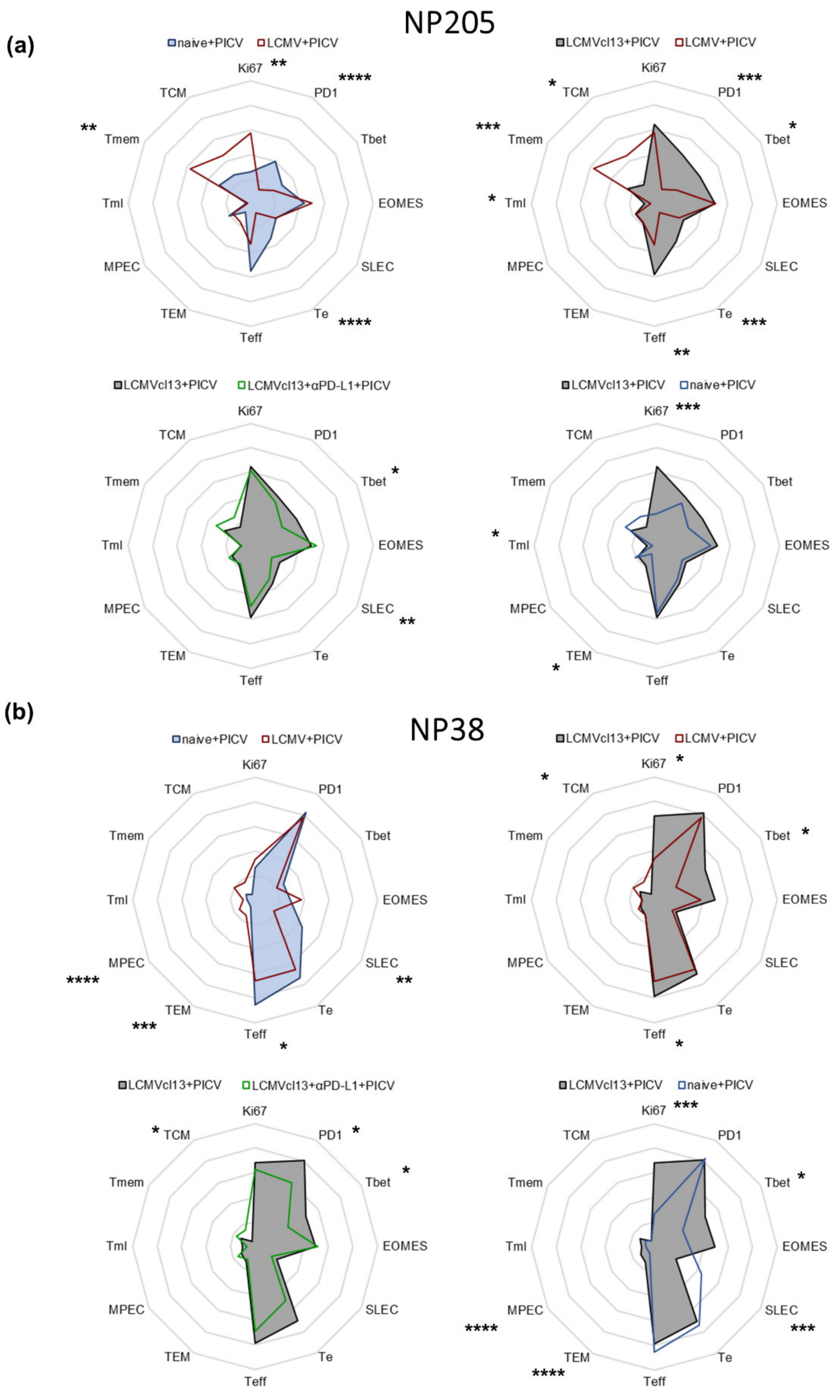

Figure S3. Phenotypical comparison of different groups from cross-reactive and non-cross-reactive T cells. Radar plots depicting the mean percentage of a) NP205- and b) NP38-Dextramer^{+} CD8^{+} T cells. Statistical comparisons are depicted with asterisks. *p=0.05; **p<0.01; ***p<0.001; ****p<0.001 (Student t-test comparison). Results are pooled from four independent experiments with n=6-11 mice/group. Each line indicates 20 % frequency increase from zero (center point) to 100 % (most outer ring). SLEC: short lived effector T cells CD127^{-}KLRG1^{+}), T_{e}: effector/terminally exhausted T cells (PD1^{+}CD127^{-}), T_{eff}: effector T cells (CD62L^{-}CD127^{-}), T_{EM}: effector-memory T cells (CD62L^{-}CD127^{+}), MPEC: memory precursor T cells (KLRG1^{-}CD127^{+}), T_{ml}: memory-like T cells (PD1^{+}CD127^{+}), T_{mem}: memory T cells (PD1^{-}CD127^{+}), T_{CM}: central memory T cells (CD62L^{+}CD127^{+}).

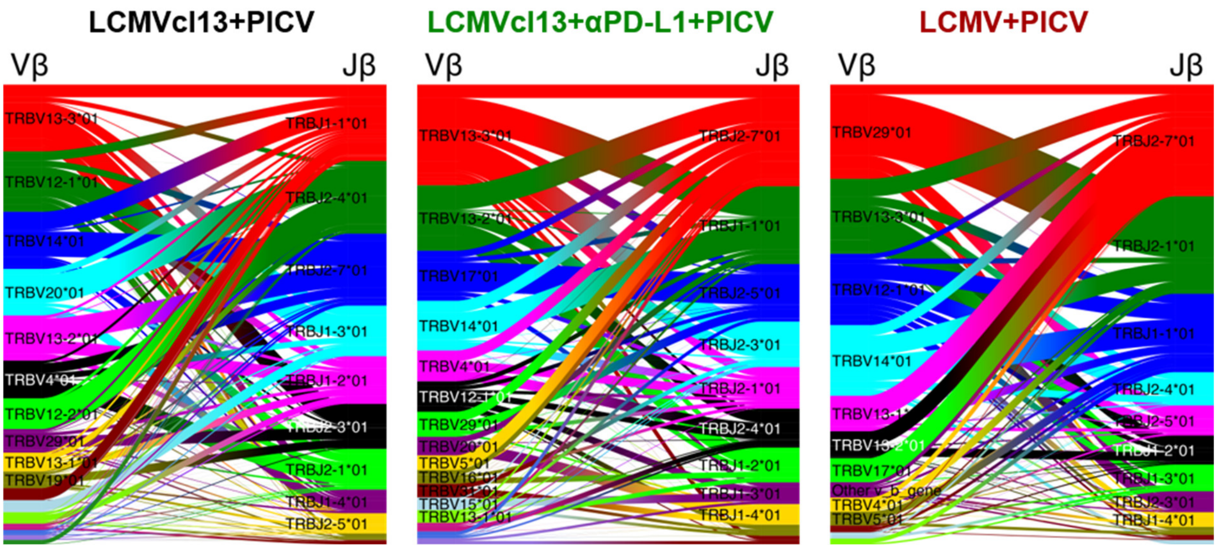

Figure S4. Vβ and Jβ usage of PICV NP38-specific T cells. Vβ and Jβ matching within the NP38-specific T cell response is depicted by lines connecting both chains, with colors from top to bottom referring to relative abundance within each individual group (ribbon plots; taking clonotype frequencies into account).
